# Magnesium Attenuates the Association Between Mixed Element Exposure and Depressive Symptoms in Older Adults

**DOI:** 10.3390/toxics14070592

**Published:** 2026-07-05

**Authors:** Dewei Shi, Fangwen Cai, Chi Zhang, Yao Xiao, Dongjia Lu, Qunan Wang

**Affiliations:** 1 Department of Toxicology, School of Public Health, Anhui Medical University, Hefei 230032, China; 2345010591@stu.ahmu.edu.cn (D.S.); 2345010567@stu.ahmu.edu.cn (F.C.); 2445010574@stu.ahmu.edu.cn (C.Z.); 2445010571@stu.ahmu.edu.cn (Y.X.); 2545010612@stu.ahmu.edu.cn (D.L.); 2 Key Laboratory of Environmental Toxicology of Anhui Higher Education Institutes, Hefei 230032, China

**Keywords:** elements, depressive symptoms, older adults, element mixture, magnesium

## Abstract

Depressive symptoms are common in older adults and may be influenced by environmental element exposure. This study evaluated associations of individual and mixed blood element concentrations with depressive symptoms in older adults. A total of 1302 community-dwelling adults aged ≥60 years were included in a 2022 cross-sectional survey, and 384 of them also participated in a 2016 survey. Whole-blood concentrations of 18 elements were measured by inductively coupled plasma mass spectrometry, and depressive symptoms were assessed using the GDS-30. Logistic regression, restricted cubic spline regression, weighted quantile sum regression, quantile g-computation, and Bayesian kernel machine regression were applied. In the longitudinal subcohort, baseline aluminum and vanadium concentrations were inversely associated with the remission of depressive symptoms. In the cross-sectional analysis, aluminum, strontium, and barium were positively associated with depressive symptoms, whereas magnesium was inversely associated. Mixture analyses suggested that the positive association of the aluminum–strontium–barium mixture with depressive symptoms was attenuated after magnesium was added to the model. These findings indicate that co-exposure to aluminum, strontium, and barium was positively associated with depressive symptoms, whereas magnesium was inversely associated and appeared to attenuate the observed mixture association.

## 1. Introduction

China became an aging society in 2021, with adults aged 65 years and older accounting for more than 14% of the total population [1]. This proportion is expected to continue rising rapidly, creating a substantial public health burden [2,3]. Among the health problems affecting older adults, depressive symptoms are particularly important but often underrecognized and undertreated. Compared with major depressive disorder, depressive symptoms are more common in later life and are associated with functional decline, poor quality of life, disease progression, disability, family disruption, and increased mortality [4,5].

Accumulating environmental evidence suggests that exposure to elements may be associated with adverse mental health outcomes, including depressive symptoms [6,7,8]. Elements, including both essential and non-essential types, are widely present in the environment and often coexist in soil, water, food, and air as a result of both natural processes and human activities [9,10,11]. Human exposure is therefore continuous and unavoidable through multiple pathways, including diet, drinking water, inhalation, and dermal contact [11]. Magnesium is an essential element involved in neurotransmitter regulation, synaptic transmission, and oxidative stress homeostasis, and abnormal magnesium status has been linked to depressive symptoms [12,13,14,15]. By contrast, aluminum (Al) may exert neurotoxic effects through oxidative stress, neuroinflammation, mitochondrial dysfunction, and disruption of neurotransmission, even after long-term low-dose exposure [16,17]. In our previous study, the non-essential elements strontium (Sr) and barium (Ba) were also associated with depressive symptoms [18].

However, most previous studies have focused on single elements or only a small number of co-exposures. Because elements may interact synergistically or antagonistically, mixture-based approaches are needed to better characterize their combined health effects [19,20,21]. Therefore, the present study aimed to investigate the associations of individual and combined blood element concentrations with depressive symptoms in older adults, with particular attention to the role of magnesium in the observed mixture associations.

## 2. Materials and Methods

### 2.1. Study Population

This study used data from a community-based cross-sectional survey conducted in Jin’an and Yu’an Districts, Lu’an City, Anhui Province, China, from July to September 2022. The survey was jointly organized by the School of Public Health, Anhui Medical University, and the Lu’an Center for Disease Control and Prevention. Participants were recruited using a probability proportional to size (PPS) sampling method.

As shown in Figure S1, 1451 community-dwelling older adults completed the questionnaire survey in 2022. After excluding 50 participants with missing whole-blood samples and 99 participants with incomplete covariate information, 1302 individuals were included in the final cross-sectional analysis. The inclusion criteria were: (1) age ≥ 60 years; (2) local residence for at least 6 months; and (3) absence of severe neuropsychiatric disorders and willingness to participate in the study. The exclusion criteria were: (1) refusal to participate in the household survey; (2) mobility or communication impairments; and (3) intellectual or psychiatric disorders that precluded effective interviewing.

Among the 1302 participants, 384 had also completed the same survey in 2016. Of these, 278 participants were free of depressive symptoms at baseline and had complete blood element measurements at both time points; they were included in the longitudinal subcohort to assess incident depressive symptoms. In addition, 106 participants who had depressive symptoms at baseline were used to evaluate remission. The study was approved by the Medical Ethics Committee of Anhui Medical University, and all participants or their legal representatives provided written informed consent.

### 2.2. Assessment of Depressive Symptoms

Depressive symptoms were assessed using the 30-item Geriatric Depression Scale (GDS-30) through face-to-face interviews. The GDS-30 is a widely used screening instrument for depression in older adults. Each item is scored as 0 or 1, yielding a total score ranging from 0 to 30. In this study, a GDS-30 score of 0–10 was considered normal, whereas a score of 11–30 indicated depressive symptoms [22].

### 2.3. Measurement of Elements

After an overnight fast of at least 8 h, venous blood samples were collected the following morning by trained hospital staff using anticoagulant vacuum tubes. A 2 mL aliquot of whole blood was transferred into labeled polypropylene (EP) tubes and stored at −80 °C until analysis. Blood concentrations of 18 elements, including Mg, Al, V, Cr, Mn, Fe, Co, Ni, Cu, Zn, As, Se, Sr, Mo, Cd, Ba, Tl, and Pb, were measured by inductively coupled plasma mass spectrometry (ICP-MS; PerkinElmer NexION 350X, PerkinElmer, Shelton, CT, USA) [23].

For sample preparation, blood samples were thawed overnight from −80 °C to −20 °C and thoroughly mixed before analysis. A 0.5 g aliquot of whole blood was placed into a 50 mL polypropylene centrifuge tube, and a reagent mixture containing Triton X-100, nitric acid, methanol, and gold solution was added to digest organic components and release inorganic ions. The sample was then adjusted to a final weight of 5.0000 g, vortexed, sealed with a paraffin membrane, and incubated at room temperature for 30 min before ICP-MS analysis. Concentrations below the limit of detection (LOD) were imputed as LOD/√2 [24]. The linearity, LODs, and LOQs of the 18 elements are presented in Table S1. The daily working conditions of the ICP-MS NexION 350X are presented in Table S2.

### 2.4. Statistical Analysis

Continuous variables are presented as mean ± standard deviation (SD) and were compared using the *t*-test. Categorical variables are presented as numbers (percentages) and were compared using the chi-square test. Blood element concentrations were non-normally distributed according to the Kolmogorov–Smirnov test and are therefore presented as median (interquartile range, IQR); comparisons were performed using the Mann–Whitney U test. Natural logarithmic (ln) transformation was applied before statistical and correlation analyses. First, logistic regression was used in the longitudinal subcohort to examine the associations between baseline element concentrations in 2016 and depressive outcomes in 2022. Odds ratios (ORs) and corresponding 95% confidence intervals (CIs) were estimated in progressively adjusted models. Next, logistic regression models were fitted to the cross-sectional data to evaluate the associations of individual elements with depressive symptoms. Elements significantly associated with depressive symptoms were retained for subsequent analyses.

Weighted quantile sum (WQS) regression was then applied to evaluate the association of element mixtures with depressive symptoms [25]. Because WQS assumes that all components in a mixture are associated with the outcome in the same direction, only Al, Sr, and Ba, which showed consistent positive associations in the single-element analyses, were included in the positive WQS model. Mg was not included in the WQS model because its association with depressive symptoms was in the opposite direction. The data were randomly divided into a training set and a validation set in a 40:60 ratio. Element weights were estimated from 3000 bootstrap iterations in the training set, and the resulting WQS index was interpreted as the association of a one-quartile increase in the element mixture with depressive symptoms.

We used a Markov chain Monte Carlo algorithm with 10,000 iterations to fit the BKMR model. The overall effect of all elements on depression was estimated by comparing the values of the exposure–response function when all exposures were at specific quantiles versus when all of them were at their median levels [26].

In addition, sensitivity analyses were performed to assess robustness. Because WQS regression assumes a consistent direction of association across mixture components, quantile g-computation (qgcomp) was also used [27]. Unlike WQS, qgcomp does not require prespecification of the direction of effect and allows both positive and negative weights. Restricted cubic spline (RCS) regression was further performed with three knots selected according to the Akaike information criterion (AIC) to examine dose–response relationships and potential non-linearity [28,29].

In addition, interactions between Mg and Al, Sr, and Ba were evaluated on both additive and multiplicative scales. Additive interaction was assessed by dichotomizing each element at the median and estimating the relative excess risk due to interaction (RERI), attributable proportion (AP), and synergy index (S). Multiplicative interaction was evaluated by including a product term (Mg × element) in multivariable logistic regression models.

To further evaluate the robustness of the observed associations, models were additionally adjusted for dietary factors, physical activity, cognitive function, medication use, serum creatinine, and white blood cell count in sensitivity analyses. The Benjamini–Hochberg false discovery rate (BH-FDR) procedure was used to account for multiple comparisons across the 18 elements.

All analyses were conducted in R software (version 4.4.0) using the packages “qgcomp”, “bkmr”, and “rms”. Two-sided *p* values < 0.05 were considered statistically significant.

## 3. Results

### 3.1. Baseline Blood Element Concentrations and Depressive Outcomes in the Longitudinal Subcohort

In the longitudinal subcohort, Table 1 presents the associations between baseline ln-transformed element concentrations and depressive outcomes. In the incident analysis, none of the evaluated elements showed a statistically significant association with depressive symptoms at follow-up. In the remission analysis, baseline ln-transformed Al and V concentrations were inversely associated with remission of depressive symptoms, whereas Mn, As, Se, Sr, Ba, and Tl were not significantly associated with remission. Among these elements, the confidence intervals for Mn and Se were wide, suggesting substantial imprecision in the estimates. The baseline characteristics of the overall 2016 cohort and the participants who completed the 2022 follow-up are presented in Table S3.

### 3.2. Characteristics of Participants in the 2022 Cross-Sectional Survey

The baseline characteristics of the 1302 participants are shown in Table 2. The sample included 552 men and 750 women, with a mean age of 72.3 ± 5.8 years. A total of 177 participants (13.59%) met the criteria for depressive symptoms. Depressive symptoms were significantly associated with age, sex, educational level, monthly income, marital status, living alone, BMI, alcohol consumption, and diabetes (all *p* < 0.05). The observed low level of multicollinearity was consistent with previously reported findings in Chinese older adults [30]. The distribution and detection rates of the 18 blood elements are presented in Table S4, and Pearson correlation coefficients among elements are shown in Figure S3. Variance inflation factors (VIFs) were calculated to assess potential multicollinearity among the 18 elemental exposures. As shown in Table S5, all VIF values ranged from 1.257 to 2.965.

### 3.3. Blood Al, Sr, and Ba Were Positively Associated with Depressive Symptoms, Whereas Mg Was Inversely Associated

Logistic regression analyses showed that the associations between blood Al, Sr, Ba, and Mg concentrations and depressive symptoms were consistent across the three progressively adjusted models (Table 3). In the fully adjusted model, Al (OR = 1.560, 95% CI: 1.126–2.174), Sr (OR = 1.890, 95% CI: 1.278–2.791), and Ba (OR = 1.623, 95% CI: 1.081–2.427) were positively associated with depressive symptoms, whereas Mg was inversely associated (OR = 0.148, 95% CI: 0.050–0.434). When elements were categorized into quartiles, participants in the highest quartile of Al, Sr, and Ba had significantly higher odds of depressive symptoms than those in the lowest quartile, whereas higher quartiles of Mg were associated with lower odds of depressive symptoms (Table S6).

### 3.4. Dose–Response Associations Between Elements and Depressive Symptoms

**Figure 1:**
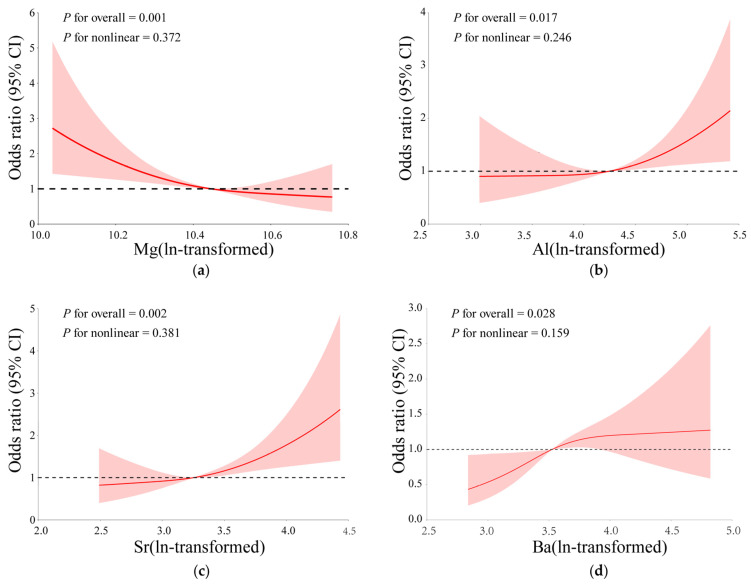
Dose–response relationships between ln-transformed blood Mg, Al, Sr, and Ba concentrations and depressive symptoms estimated by restricted cubic spline (RCS) regression. The red line represents the estimated odds ratio, and the shaded area indicates the 95% confidence interval (CI). Subfigure (**a**) shows Mg, subfigure (**b**) shows Al, subfigure (**c**) shows Sr, and subfigure (**d**) shows Ba.

RCS analysis indicated approximately linear associations for Mg, Al, Sr, and Ba [28,29]. Higher blood Al, Sr, and Ba concentrations were associated with higher odds of depressive symptoms, whereas higher blood Mg concentrations were associated with lower odds of depressive symptoms (Figure 1).

### 3.5. Sex-Specific Associations of Al, Sr, Ba, and Mg with Depressive Symptoms

**Figure 2:**
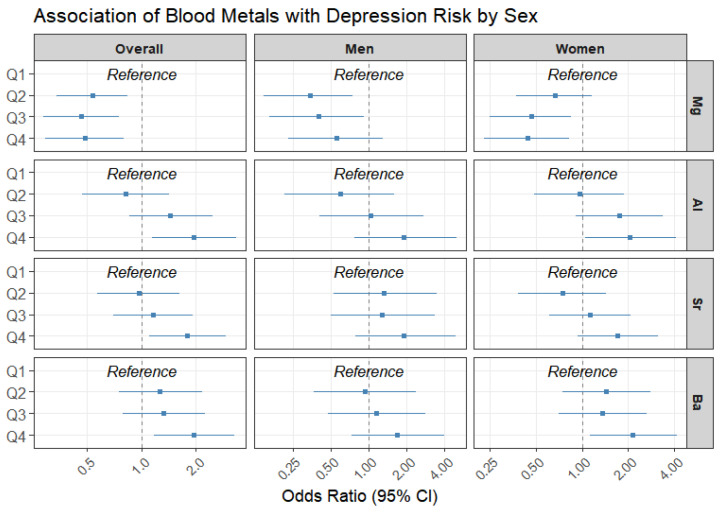
Sex-stratified associations of blood Mg, Al, Sr, and Ba concentrations with depressive symptoms.The blue lines represent the odds ratios and 95% confidence intervals (CI) for each quartile, and Q1 was used as the reference group.

Given known sex differences in element metabolism, susceptibility to neurotoxicity, and depression prevalence [31], stratified analyses were conducted by sex (Figure 2). Among women, the highest quartiles of Al and Ba were significantly positively associated with depressive symptoms compared with the lowest quartile. As a continuous variable, Sr was positively associated with depressive symptoms in women but not in men. By contrast, Mg remained inversely associated with depressive symptoms in the overall population.

### 3.6. Combined Exposure to Al, Sr, and Ba Was Positively Associated with Depressive Symptoms, and This Association Was Attenuated by Mg

As shown in Figure S3, in the WQS model adjusted for all covariates, the WQS index comprising Al, Sr, and Ba was significantly positively associated with depressive symptoms (OR = 1.394, 95% CI: 1.082–1.791), suggesting that the Al–Sr–Ba mixture was associated with depressive symptoms. Among the mixture components, Al contributed the largest weight. In the qgcomp sensitivity analysis, the combined exposure remained significantly associated with depressive symptoms (OR = 1.466, 95% CI: 1.183–1.816), again with Al contributing the greatest weight.

**Figure 3:**
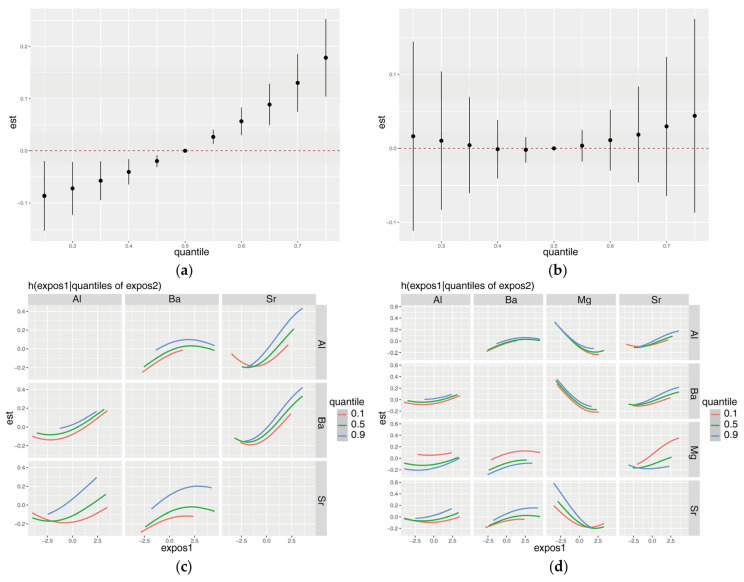
Bayesian kernel machine regression (BKMR) analysis of the associations between element mixture exposure and depressive symptoms and pairwise interactions among elements. The black dots represent the estimated effects, and the vertical lines indicate the corresponding 95% confidence intervals (CI). (**a**) Estimated overall effect of the Al–Sr–Ba mixture on depressive symptoms; (**b**) estimated overall effect after Mg was added to the mixture; (**c**) pairwise exposure–response relationships among Al, Ba, and Sr; (**d**) pairwise exposure–response relationships among Al, Ba, Mg, and Sr. Red, green, and blue lines indicate the exposure–response curves when the column element is fixed at the 10th, 50th, and 90th percentiles, respectively.

In the BKMR analysis of the three-element mixture, exposures at or above the 55th percentile were associated with a significantly higher risk of depressive symptoms compared with the 50th percentile. Notably, when Mg was added to the mixture, the positive association was attenuated to null in the statistical model (Figure 3). As illustrated in Figure 3, the slope of the dose–response association between Sr and depressive symptoms depended on Mg concentration (10th, 50th, and 90th percentiles), suggesting a possible interaction between the two elements.

We further assessed interactions between Mg and Al, Sr, and Ba on additive and multiplicative scales (Table 4). No significant interaction was observed for Mg with Al or Ba. For Mg and Sr, negative additive interaction was suggested by the RERI and AP estimates, whereas no significant multiplicative interaction was detected.

### 3.7. Sensitivity Analysis

To evaluate the robustness of the primary findings, the multivariable logistic regression models were additionally adjusted for dietary factors, physical activity, cognitive function, medication use, serum creatinine, and white blood cell count. The Benjamini–Hochberg false discovery rate (BH-FDR) procedure was then applied to account for multiple comparisons across the 18 elements. As shown in Table 5, the inverse association between Mg and depressive symptoms remained statistically significant after BH-FDR correction (OR = 0.169, 95% CI: 0.057–0.502; q = 0.025), and Sr remained positively associated with depressive symptoms (OR = 1.767, 95% CI: 1.190–2.623; q = 0.043). Al and Ba showed borderline associations after correction (q = 0.067 for both), whereas no statistically significant associations were observed for the remaining elements. These findings were broadly consistent with the primary analysis and support the robustness of the observed associations.

## 4. Discussion

Using cross-sectional data and a 6-year longitudinal subcohort of community-dwelling older adults in Lu’an City, Anhui Province, this study systematically examined the associations between multiple element exposures and depressive symptoms. The main findings were as follows: (1) no baseline element concentration was significantly associated with incident depressive symptoms in the longitudinal subcohort, whereas baseline Al and V were inversely associated with remission; (2) Al, Sr, and Ba were positively and approximately linearly associated with depressive symptoms, whereas Mg showed an inverse association; (3) mixture analyses indicated that co-exposure to Al, Sr, and Ba was positively associated with depressive symptoms, and this association was attenuated to null after Mg was added; and (4) these associations were stronger in women than in men.

To our knowledge, this study is among the first to examine longitudinal associations between baseline blood element concentrations and depressive symptom outcomes in older adults. Previous studies have reported associations between Sr, Ba, and depressive symptoms, particularly among older women, but temporal evidence has been limited [15,32]. In the present study, no baseline element concentration was significantly associated with incident depressive symptoms at follow-up. In the remission analysis, baseline Al and V concentrations were inversely associated with remission of depressive symptoms, although these findings should be interpreted cautiously given the small sample size for remission. These results suggest that baseline blood element concentrations may not robustly predict incident depressive symptoms over 6 years in this cohort, whereas some associations with remission were observed and require further confirmation.

Aluminum is a non-essential element widely distributed in the environment and is increasingly recognized as potentially neurotoxic [17]. Epidemiological evidence has suggested that individuals with chronic occupational or environmental aluminum exposure may have a higher prevalence of depressive symptoms, and the risk may increase with exposure level [33]. Experimental studies indicate that aluminum may impair dopamine synthesis, disrupt neurotransmitter homeostasis, interfere with mitochondrial energy metabolism, and induce oxidative stress and neuronal dysfunction [34,35]. These mechanisms may help explain the positive associations observed in the present study.

Our group previously reported that blood Sr and Ba were positively associated with depressive symptoms in older women [18]. More recently, Sun et al. reported that serum Ba levels were positively associated with depression scale scores across quantiles [32]. The neurotoxic potential of Sr and Ba, both alkaline earth elements, may be related to disruption of calcium signaling. These elements may compete with Ca^2+^ and Mg^2+^ in biological systems, interfere with calcium-dependent signaling pathways, and alter neuronal excitability through their effects on voltage-gated calcium channels [36,37]. Such disturbances may affect cholinergic and monoaminergic neurotransmission and contribute to dysfunction in neural circuits involved in mood regulation.

In contrast, Mg showed a clear inverse association with depressive symptoms. Magnesium is essential for neuronal function and the maintenance of brain homeostasis [38]. Previous observational studies and meta-analyses have shown that magnesium intake or serum magnesium status is inversely associated with depression risk [12,13], and magnesium deficiency has been linked to depressive-like behaviors in animal models [14,15]. Mechanistically, Mg^2+^ may compete with Sr^2+^ and Ba^2+^ for entry through calcium channels, thereby limiting the transmembrane influx of these elements and reducing their potential neurotoxic effects [39]. The attenuation of the positive association between the Al–Sr–Ba mixture and depressive symptoms after Mg was added should be interpreted as a model-based statistical finding rather than evidence of biological antagonism. Given the correlations among elements and the observational design, this attenuation may reflect shared exposure patterns, reallocation of model weights, or residual confounding. In addition, formal interaction analyses were conducted on both additive and multiplicative scales for Mg with Al, Sr, and Ba. Table 4 suggested a negative additive interaction between Mg and Sr. This means that the combined association of high Mg and high Sr exposure with depressive symptoms was less than what would be expected under simple additivity, which may indicate a protective modifying role of Mg.

The sex-specific findings observed in this study may reflect differences in element metabolism, hormonal status, and susceptibility to neurotoxic insults. Women may be more vulnerable to the adverse mental health effects of certain element exposures, potentially because of sex-related differences in body composition, iron status, endocrine factors, and inflammatory responses [31]. This may help explain why the associations of Al, Sr, and Ba with depressive symptoms were more apparent in women.

This study has several strengths. First, it included a relatively large community-based sample of older adults and comprehensively adjusted for multiple potential confounders. Second, 18 blood elements were measured, allowing both single-element and mixture-based analyses. Third, depressive symptoms were assessed using a validated screening instrument (GDS-30). Fourth, longitudinal data on baseline blood element concentrations and follow-up depressive outcomes were available for a subcohort, enabling the examination of incident symptoms and remission. Finally, a range of advanced statistical methods, including WQS, qgcomp, BKMR, and RCS, were used to evaluate mixture effects and potential non-linearity.

This study has several limitations. First, the cross-sectional design precludes causal inference. Second, residual confounding may still exist despite adjustment for multiple covariates. Third, the longitudinal subcohort was relatively small, particularly for the remission analysis, which may have limited statistical precision. Finally, the findings from older adults in one Chinese city may not be directly generalizable to other populations.

## 5. Conclusions

This study suggests that higher blood Mg concentrations are associated with lower odds of depressive symptoms, whereas elevated Al, Sr, and Ba concentrations are associated with higher odds. In the statistical models, adding Mg weakened the association between the Al–Sr–Ba mixture and depressive symptoms, and a negative additive interaction was observed for Mg and Sr.

## Figures and Tables

**Table 1:** Associations between baseline blood element concentrations in 2016 and depressive symptoms in 2022 in the longitudinal subcohort.

Elements (μg/L)	Incident of Depression Symptoms (N = 278)		Remission of Depressive Symptoms (N = 106)	
	OR (95% CI)	*p* Value	OR (95% CI)	*p* Value
Al	0.852 (0.551, 1.320)	0.474	0.518 (0.289, 0.928)	0.027
V	1.009 (0.585, 1.739)	0.975	0.243 (0.077, 0.769)	0.016
Mn	0.678 (0.089, 5.146)	0.707	0.666 (0.044, 10.139)	0.770
As	0.656 (0.314, 1.370)	0.262	1.256 (0.463, 3.408)	0.655
Se	0.931 (0.124, 6.975)	0.945	0.293 (0.011, 7.896)	0.465
Sr	1.183 (0.378, 3.702)	0.773	0.371 (0.056, 2.470)	0.306
Ba	1.232 (0.548, 2.769)	0.613	0.846 (0.283, 2.533)	0.765
Tl	1.161 (0.702, 1.917)	0.561	0.527 (0.213, 1.304)	0.166

Notes: Element concentrations were natural log-transformed before analysis. Logistic regression models were used to estimate odds ratios (ORs) and 95% confidence intervals (CIs) for incident depressive symptoms among participants free of depressive symptoms at baseline and for remission of depressive symptoms among participants with depressive symptoms at baseline. ORs are presented per one-unit increase in ln-transformed element concentration.

**Table 2:** Baseline characteristics of participants in the 2022 cross-sectional survey.

Characteristics	Total Participants	Depressive Symptoms		*p* Value
		No	Yes	
Age	72.3 ±5.8	73.9 ± 6.50	72 ± 5.6	0.002
Sex				
Male	552 (42.40%)	489 (43.47%)	63 (35.59%)	0.048
Female	750 (57.60%)	636 (56.53%)	114 (64.41%)	
Education				
Junior high school and below	1165 (89.48%)	995 (88.44%)	170 (96.05%)	0.009
Senior high school	99 (7.60%)	94 (8.36%)	5 (2.82%)	
College and above	38 (2.92%)	36 (3.20%)	2 (1.13%)	
Income				
Less than 1000 yuan	528 (40.55%)	440 (39.11%)	88 (49.72%)	<0.001
1001–2000 yuan	248 (19.05%)	203 (18.04%)	45 (25.42%)	
2001–3000 yuan	286 (21.97%)	263 (23.38%)	23 (12.99%)	
More than 3001 yuan	240 (18.43%)	219 (19.47%)	21 (11.86%)	
Marital status				
Never married	9 (0.69%)	5 (0.44%)	4 (2.26%)	<0.001
Currently married	1022 (78.49%)	912 (81.07%)	110 (62.15%)	
Previously married	271 (20.81%)	208 (18.49%)	63 (35.59%)	
Living alone				
Yes	179 (13.75%)	138 (12.27%)	41 (23.16%)	<0.001
No	1123 (86.25%)	987 (87.73%)	136 (76.84%)	
BMI (kg/m^2^)				
<18.5	57 (4.38%)	40 (3.56%)	17 (9.60%)	0.002
18.5 ≤ BMI < 24.0	593 (45.55%)	513 (45.60%)	80 (45.20%)	
24.0 ≤ BMI < 28.0	482 (37.02%)	419 (37.24%)	63 (35.59%)	
≥28.0	170 (13.06%)	153 (13.60%)	17 (9.60%)	
Smoking				
Never smoked	1019 (78.26%)	876 (77.87%)	143 (80.79%)	0.234
Former smoker	191 (14.67%)	172 (15.29%)	19 (10.73%)	
Current smoker	92 (7.07%)	77 (6.84%)	15 (8.47%)	
Drinking				
Never drank	876 (67.28%)	746 (66.31%)	130 (73.45%)	0.037
Former drinker	351 (26.96%)	317 (28.18%)	34 (19.21%)	
Current drinker	75 (5.76%)	62 (5.51%)	13 (7.34%)	
Diabetes				
Yes	207 (15.90%)	169 (15.02%)	38 (21.47%)	0.029
No	1095 (84.10%)	956 (84.98%)	139 (78.53%)	
Hypertension				
Yes	700 (53.76%)	599 (53.24%)	101 (57.06%)	0.344
No	602 (46.24%)	526 (46.76%)	76 (42.94%)	
Coronary heart disease				
Yes	128 (9.83%)	107 (9.51%)	21 (11.86%)	0.328
No	1174 (90.17%)	1018 (90.49%)	156 (88.14%)	
MMSE scores				
	23.0 (5.4)	19.5 (6.1)	22.5 (5.6)	<0.001
Serum creatinine				
	71.1 (23.2)	72.1 (29.8)	71.2 (24.2)	0.866
WBC				
	5.8 (1.4)	5.6 (1.4)	5.8 (1.4)	0.130
Balanced diet				
Yes	596 (52.98%)	66 (37.29%)	662 (50.84%)	<0.001
No	529 (47.02%)	111 (62.71%)	640 (49.16%)	
Physical activity				
Yes	330 (29.33%)	69 (38.98%)	399 (30.65%)	0.009
No	795 (70.67%)	108 (61.02%)	903 (69.35%)	
Gastritis medication				
Yes	46 (4.09%)	9 (5.08%)	55 (4.22%)	0.540
No	1079 (95.91%)	168 (94.92%)	1247 (95.78%)	

Notes: Data are presented as mean ± SD for continuous variables and as counts (percentages) for categorical variables. MMSE = Mini-Mental State Examination; higher scores indicate better cognitive function. *p* values compare the no depressive symptoms group with the yes depressive symptoms group and were derived from the appropriate tests: Student’s *t*-test for normally distributed continuous variables, Wilcoxon rank-sum test for non-normally distributed continuous variables, and Chi-square or Fisher’s exact test for categorical variables. Abbreviations: BMI = body mass index; WBC = white blood cell count.

**Table 3:** Associations between ln-transformed blood element concentrations and depressive symptoms in multivariable logistic regression models.

Elements	Model I		Model II		Model III	
	OR (95% CI)	*p* Value	OR (95% CI)	*p* Value	OR (95% CI)	*p* Value
Mg	0.138 (0.047–0.397)	<0.001	0.148 (0.050–0.434)	0.001	0.148 (0.050–0.434)	0.001
Al	1.568 (1.137–2.176)	0.007	1.560 (1.124–2.179)	0.008	1.560 (1.126–2.174)	0.008
V	1.125 (0.975–1.316)	0.122	1.120 (0.967–1.318)	0.148	1.101 (0.950–1.294)	0.222
Cr	1.028 (0.940–1.132)	0.559	1.029 (0.939–1.135)	0.554	1.044 (0.952–1.153)	0.371
Mn	1.208 (0.844–1.770)	0.316	1.130 (0.785–1.669)	0.523	1.174 (0.814–1.735)	0.404
Fe	0.898 (0.308–2.627)	0.843	0.831 (0.280–2.473)	0.738	0.810 (0.271–2.430)	0.706
Co	1.025 (0.952–1.111)	0.526	1.018 (0.945–1.104)	0.65	1.015 (0.942–1.100)	0.707
Ni	0.874 (0.747–1.026)	0.097	0.861 (0.734–1.013)	0.069	0.869 (0.741–1.022)	0.086
Cu	3.134 (0.908–10.949)	0.072	2.933 (0.831–10.475)	0.096	2.937 (0.828–10.549)	0.097
Zn	0.984 (0.518–1.881)	0.961	0.981 (0.510–1.897)	0.955	0.974 (0.507–1.881)	0.937
As	1.092 (0.893–1.368)	0.686	1.065 (0.866–1.338)	0.784	1.068 (0.867–1.344)	0.787
Se	0.812 (0.553–1.204)	0.29	0.829 (0.562–1.228)	0.342	0.841 (0.570–1.246)	0.379
Sr	1.896 (1.291–2.782)	0.001	1.878 (1.272–2.772)	0.002	1.890 (1.278–2.791)	0.001
Mo	1.183 (0.874–1.647)	0.301	1.123 (0.827–1.574)	0.482	1.108 (0.816–1.551)	0.533
Cd	0.872 (0.717–1.072)	0.181	0.872 (0.717–1.072)	0.181	0.887 (0.727–1.093)	0.248
Ba	1.612 (1.084–2.386)	0.017	1.648 (1.098–2.464)	0.015	1.623 (1.081–2.427)	0.019
Tl	0.938 (0.871–1.013)	0.098	0.942 (0.873–1.019)	0.127	0.940 (0.871–1.017)	0.116
Pb	1.008 (0.925–1.104)	0.860	0.999 (0.916–1.094)	0.975	1.002 (0.918–1.099)	0.967

Notes: OR, odds ratio; CI, confidence interval. Model I: adjusted for age, sex, educational level, income, BMI, marital status, and living status. Model II: Model I + drinking and smoking. Model III: Model II + hypertension, diabetes, and coronary heart disease.

**Table 4:** Additive and multiplicative interactions between blood Mg and other elements (Al, Sr, and Ba) in relation to depressive symptoms in older adults.

	Additive Interactive				Multiplicative Interactive	
	Measure	Estimate	Lower	Upper	OR (95% CI)	*p* Value
Mg × Al	RERI	−0.017	−1.220	0.868	0.439 (0.061, 3.150)	0.413
	AP	−0.020	−0.925	0.637		
	S	−2.997	−6.141	7.866		
Mg × Sr	RERI	−2.576	−5.220	−0.961	0.147 (0.017, 1.279)	0.082
	AP	−2.829	−6.017	−1.035		
	S	−0.080	−0.685	0.238		
Mg × Ba	RERI	−0.150	−1.203	0.566	0.536 (0.051, 5.671)	0.604
	AP	−0.160	−1.245	0.611		
	S	−1.669	−6.067	7.393		

Notes: RERI, relative excess risk due to interaction; AP, attributable proportion due to interaction; S, synergy index; OR, odds ratio; CI, confidence interval. Additive interaction was assessed by dichotomizing each element at the median and estimating RERI, AP, and S. Multiplicative interaction was evaluated by including a product term (Mg × element) in multivariable logistic regression models. For additive interaction, values of RERI < 0, AP < 0, or S < 1 suggest a negative interaction, whereas values of RERI > 0, AP > 0, or S > 1 suggest a positive interaction. *p* values for multiplicative interaction refer to the product term in the logistic regression model.

**Table 5:** Sensitivity analysis of associations between 18 elements and depressive symptoms after additional covariate adjustment and BH-FDR correction.

Elements	OR (95% CI)	*p* Value	q Value
Mg	0.169 (0.057–0.502)	0.001	0.025
Al	1.512 (1.084–2.108)	0.015	0.067
V	1.088 (0.929–1.273)	0.297	0.535
Cr	1.062 (0.963–1.171)	0.226	0.452
Mn	1.147 (0.785–1.676)	0.478	0.782
Fe	0.772 (0.254–2.346)	0.648	0.832
Co	1.012 (0.936–1.094)	0.771	0.867
Ni	0.868 (0.738–1.021)	0.088	0.316
Cu	2.846 (0.789–10.265)	0.110	0.330
Zn	1.046 (0.535–2.045)	0.896	0.896
As	1.051 (0.842–1.313)	0.659	0.832
Se	0.924 (0.623–1.370)	0.693	0.832
Sr	1.767 (1.190–2.623)	0.005	0.043
Mo	1.075 (0.775–1.490)	0.666	0.832
Cd	0.868 (0.703–1.071)	0.186	0.419
Ba	1.681 (1.112–2.542)	0.014	0.067
Tl	0.945 (0.874–1.021)	0.152	0.391
Pb	0.991 (0.905–1.086)	0.853	0.896

Notes: Results are presented as odds ratios (ORs) with 95% confidence intervals (CIs). *p* values were obtained from multivariable logistic regression models. To account for multiple testing across the 18 elements, false discovery rate (FDR) correction was performed using the Benjamini–Hochberg (BH) procedure, and q values are reported. In addition to the main covariates specified in the study design, the models were further adjusted for dietary factors, physical activity, cognitive function, medication use, serum creatinine, and white blood cell count.

## Data Availability

The datasets generated and analyzed during the current study are not publicly available due to confidentiality restrictions specified in the ethics approval. However, they are available from the corresponding author upon reasonable request and subject to approval by the ethics committee.

## Supplementary Materials

The following supporting information can be downloaded at: https://www.mdpi.com/article/10.3390/toxics14070592/s1: Figure S1. Flow chart of participant selection; Figure S2. Pearson correlation heatmap for ln-transformed blood elements; Figure S3. Weights estimated from the weighted quantile sum (WQS) and quantile g-computation (qgcomp) models for depressive symptoms; Table S1. Linearity, limits of detection (LODs), and limits of quantification (LOQs) for the 18 elements; Table S2. ICP-MS NexION 350X daily working conditions; Table S3. Baseline characteristics of the 2016 cohort overall and participants who returned for the 2022 follow-up; Table S4. Distribution of blood element concentrations in the 2022 survey; Table S5. Variance inflation factors for 18 elemental exposures; Table S6. Quartile-based associations between blood element concentrations and depressive symptoms in multivariable logistic regression models.

## Abbreviations

The following abbreviations are used in this manuscript:

BKMR	Bayesian kernel machine regression
BMI	Body mass index
CI	Confidence interval
GDS-30	30-item Geriatric Depression Scale
ICP-MS	Inductively coupled plasma mass spectrometry
IQR	Interquartile range
LOD	Limit of detection
LOQ	Limit of quantification
OR	Odds ratio
PPS	Probability proportional to size
Qgcomp	Quantile g-computation
RCS	Restricted cubic spline
SD	Standard deviation
WQS	Weighted quantile sum
