# Supplementary material for: Magnesium Attenuates the Association Between Mixed Element Exposure and Depressive Symptoms in Older Adults
Source: Toxics. 2026 Jul 5;14(7):592. doi: 10.3390/toxics14070592 (PMC13417367; doi:10.3390/toxics14070592)
Supplement: Supplementary file 1 [file toxics-14-00592-s001.zip › toxics-4346650-supplementary.pdf]

Supporting information

Magnesium Attenuates the Association Between Mixed Element Exposure and Depressive Symptoms in Older Adults

Dewei Shi [1,2], Fangwen Cai [1,2], Chi Zhang [1,2], Yao Xiao [1,2], Dongjia Lu [1,2] and Qunan Wang [1,2,*]

[1] Department of Toxicology, School of Public Health, Anhui Medical University, Hefei 230032, China; 2345010591@stu.ahmu.edu.cn (D.S.); 2345010567@stu.ahmu.edu.cn (F.C.); 2445010574@stu.ahmu.edu.cn (C.Z.); 2445010571@stu.ahmu.edu.cn (Y.X.); 2545010612@stu.ahmu.edu.cn (D.L.)

[2] Key Laboratory of Environmental Toxicology of Anhui Higher Education Institutes, Hefei 230032, China

* Correspondence: wqn@ahmu.edu.cn

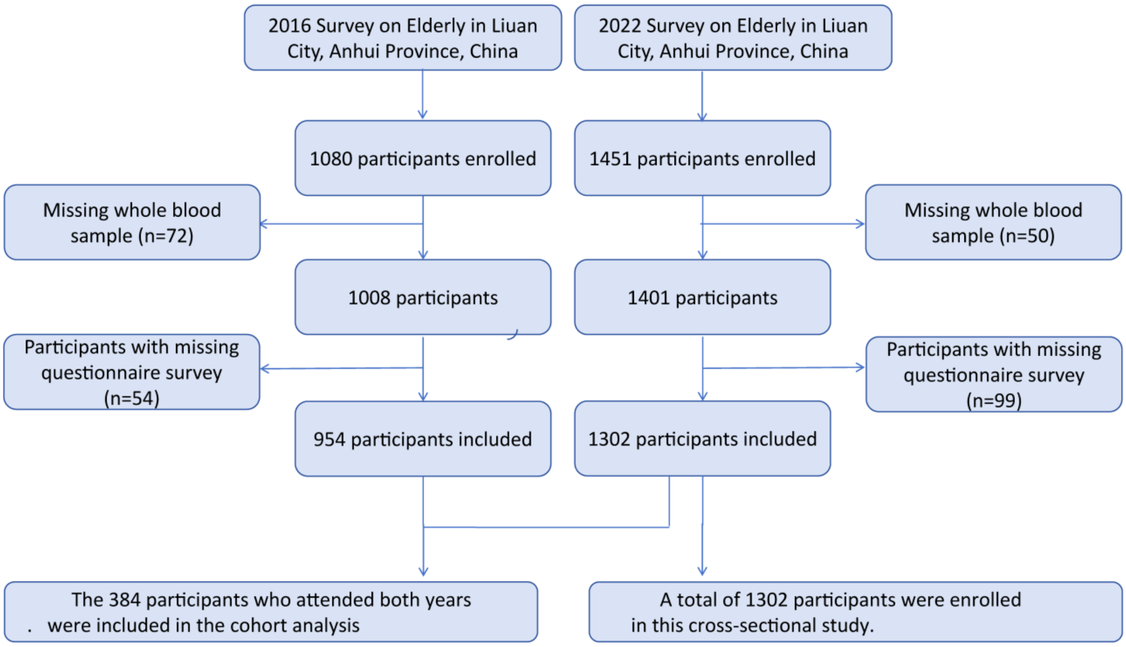

Figure S1. Flow chart of participant selection.

Table S1. Linearity, limits of detection (LODs), and limits of quantification (LOQs) for the 18 elements.

elements	Regression equation	r	LOD(μg/L)	LOQ(μg/L)
V	y=0.379x+0.000	0.999998	0.000674	0.002022
Cr	y=0.530x+0.000	0.999644	0.027433	0.082299
Mn	y=0.293x+0.000	0.999988	0.010803	0.032409
Fe	y=2.776x+0.000	0.999851	0.001141	0.003423
As	y=0.142x+0.000	0.999868	0.009968	0.029904
Se	y=0.001x+0.000	0.999755	1.090326	3.270978
Sr	y=0.070x+0.000	0.999974	0.003319	0.009957
Mo	y=0.101x+0.000	0.999997	0.002186	0.006558
Cd	y=0.016x+0.000	0.999962	0.002644	0.007932
Co	y=1.741x+0.000	0.999994	0.000284	0.000852
Ni	y=0.485x+0.000	0.999885	0.109681	0.329043
Cu	y=0.378x+0.000	0.999904	0.116873	0.350619
Zn	y=0.029x+0.000	0.999912	0.841127	2.523381
Ba	y=0.168x+0.000	0.999995	0.005989	0.017967
Tl	y=0.187x+0.000	0.999988	0.000682	0.002046
Pb	y=0.121x+0.000	0.999998	0.012816	0.038448
Mg	y=0.011x+0.000	0.999942	0.354492	1.063476
Al	y=0.013x+0.000	0.999391	0.384228	1.152684

Notes: This table presents the calibration curves, correlation coefficients, limits of detection (LODs), and limits of quantification (LOQs) for the 18 elements measured in whole blood by inductively coupled plasma mass spectrometry (ICP-MS). LOD, limit of detection; LOQ, limit of quantification; ICP-MS, inductively coupled plasma mass spectrometry.

Table S2. ICP-MS NexION 350X daily working conditions

Instrumental Parameter	Requirement
Ar nebulizer gas flow	0.90 L/min (optimized daily)
Auxiliary gas	1.2
Plasma gas	15
RF power	1100 W
Measurement units	Cps (counts per second)
Detector mode	Dual (KEDa)
Curve type Simple	linear
Auto lens	On
Blank subtraction	After internal standard
Sample units	μg/L
Sweeps/reading	20
Readings/replicate	1
Replicates	3
Dwell time	50 ms

a KED stands for kinetic energy discrimination.

Table S3. Baseline characteristics of the 2016 cohort overall and participants who returned for the 2022 follow-up.

Characteristics	Total (N=954)	Returned (n=384)	*P* value
**Age**	71.76 (7.52)	69.69 (18.15)	<0.001
**Sex**			
Male	429 (44.97)	176 (45.83)	0.821
Female	525 (55.03)	208 (54.17)	
**Education**			
Junior high school and below	843 (88.36)	352 (91.67)	0.136
Senior high school	97 (10.17)	30 (7.81)	
College and above	14 (1.47)	2 (0.52)	
**Income**			
Less than 1000 yuan	459 (48.11)	213 (55.47)	0.071
1001–2000 yuan	129 (13.52)	45 (11.72)	
2001–3000 yuan	87 (9.12)	24 (6.25)	
More than 3001 yuan	279 (29.25)	102 (26.56)	
**Marital status**			
Never married	14 (1.47)	2 (0.52)	0.028
Currently married	682 (71.49)	300 (78.12)	
Previously married	258 (27.04)	82 (21.35)	
**Living alone**			
Yes	132 (13.84)	34 (8.85)	0.016
No	822 (86.16)	350 (91.15)	
**BMI（kg/m²）**			
<18.5	48 (5.03)	14 (3.65)	0.743
18.5≤BMI<24.0	413 (43.29)	171 (44.53)	
24.0≤BMI<28.0	350 (36.69)	142 (36.98)	
≥28.0	143 (14.99)	57 (14.84)	
**Smoking**			
Never smoked	769 (80.61)	310 (80.73)	0.702
Former smoker	34 (3.56)	17 (4.43)	
Current smoker	151 (15.83)	57 (14.84)	
**Drinking**			
Never drank	606 (63.52)	216 (56.25)	0.041
Former drinker	86 (9.01)	45 (11.72)	
Current drinker	262 (27.46)	123 (32.03)	
**Diabetes**			
Yes	142 (14.88)	48 (12.50)	0.297
No	812 (85.12)	336 (87.50)	
**Hypertension**			
Yes	489 (51.26)	184 (47.92)	0.296
No	465 (48.74)	200 (52.08)	

**Coronary heart disease**			
Yes	127 (13.31)	45 (11.72)	0.485
No	827 (86.69)	339 (88.28)	

Notes: Values are presented as mean (SD) or number (percentage). P values were calculated using the t-test for continuous variables and the chi-square test for categorical variables. BMI, body mass index.

Table S4. Distribution of blood element concentrations in the 2022 survey.

elements (μg/L)	DF	Mean±SD	GM	Median	Percentiles				
					P5	P25	P50	P75	P95
Mg	100.00%	34.249 ± 5.352	33.824	34.411	25.493	30.310	34.411	37.914	42.499
Al	100.00%	85.547 ± 51.169	72.477	69.786	29.218	47.287	69.786	117.736	184.166
V	94.50%	0.445 ± 0.331	0.303	0.367	0.005	0.260	0.367	0.543	0.988
Cr	88.00%	5.876 ± 13.264	2.284	4.177	0.019	2.726	4.177	6.166	10.290
Mn	100.00%	46.696 ± 16.996	42.413	47.014	12.789	40.611	47.014	57.240	71.053
Fe	100.00%	0.447 ± 0.07	0.441	0.440	0.349	0.403	0.440	0.480	0.583
Co	88.00%	0.189 ± 0.271	0.071	0.149	0.000	0.095	0.149	0.210	0.407
Ni	95.50%	3.104 ± 6.662	1.701	1.825	0.192	1.159	1.825	3.124	7.553
Cu	100.00%	0.866 ± 0.118	0.858	0.863	0.691	0.786	0.863	0.933	1.056
Zn	100.00%	7.520 ± 1.947	7.282	7.362	4.510	6.431	7.362	8.355	10.742
As	99.00%	1.384 ± 1.482	1.082	1.162	0.389	0.812	1.162	1.617	2.910
Se	99.99%	0.114 ± 0.052	0.105	0.107	0.058	0.083	0.107	0.134	0.175
Sr	100.00%	30.744 ± 15.411	27.866	25.865	15.886	20.580	25.865	35.255	63.468
Mo	99.90%	1.153 ± 0.628	0.995	1.030	0.412	0.699	1.030	1.458	2.354
Cd	99.80%	1.436 ± 1.462	0.990	0.975	0.242	0.607	0.975	1.680	4.050
Ba	100.00%	39.638 ± 21.588	36.057	33.943	20.819	26.516	33.943	46.395	70.439
Tl	80.80%	0.064 ± 0.051	0.024	0.060	0.000	0.017	0.060	0.094	0.154
Pb	85.40%	28.085 ± 111.976	9.104	18.483	0.070	12.440	18.483	25.624	42.632

Notes: Values for Mg, Fe, Cu, Zn, and Se are multiplied by 10^3 .

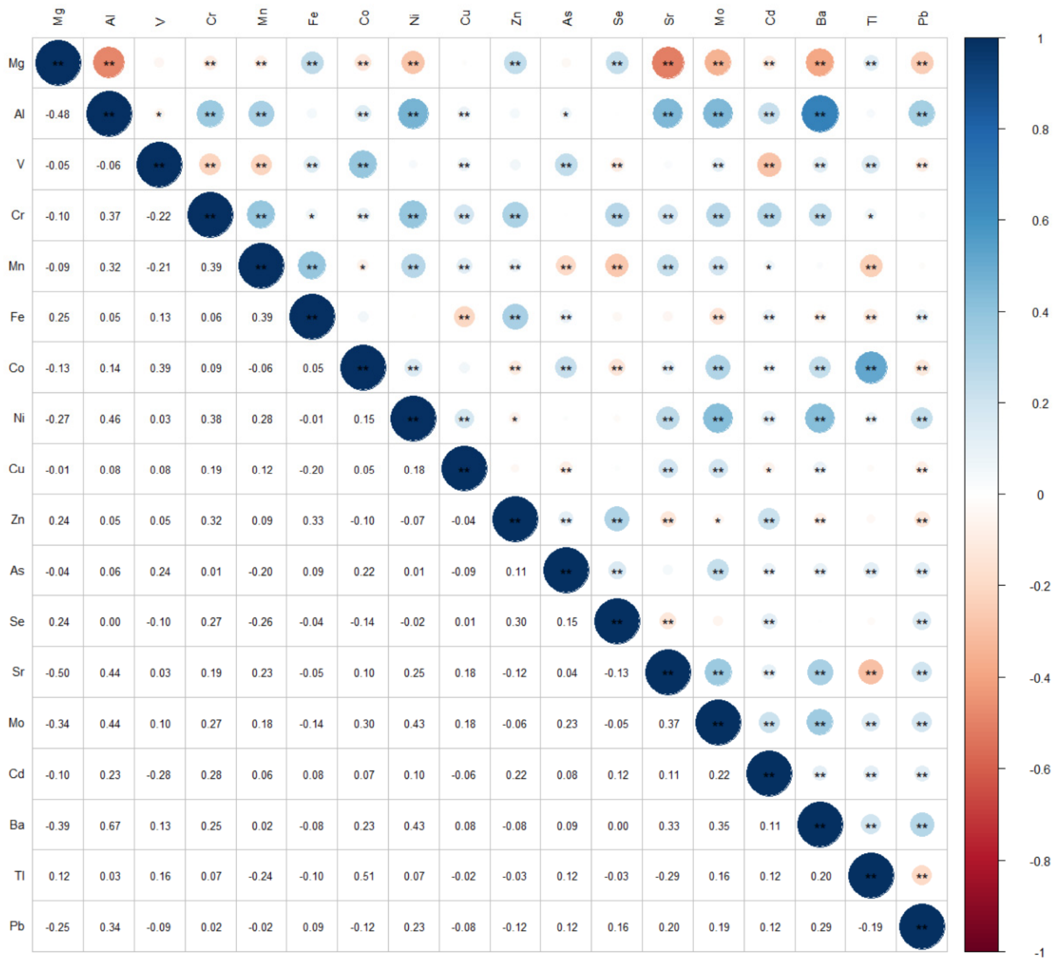

Figure S2. Pearson correlation heatmap for ln-transformed blood elements.

Table S5. Variance inflation factors for 18 elemental exposures.

Variable	VIF
Mg	2.051
Al	2.965
V	1.845
Cr	2.159
Mn	2.553
Fe	2.063
Co	1.931
Ni	1.594
Cu	1.257
Zn	1.688
As	1.289
Se	1.687
Sr	1.83
Mo	1.79
Cd	1.407
Ba	2.287
Tl	2.072
Pb	1.545

Notes: Variance inflation factors (VIFs) were calculated for 18 elements: Magnesium (Mg), Aluminum (Al), Vanadium (V), Chromium (Cr), Manganese (Mn), Iron (Fe), Cobalt (Co), Nickel (Ni), Copper (Cu), Zinc (Zn), Arsenic (As), Selenium (Se), Strontium (Sr), Molybdenum (Mo), Cadmium (Cd), Barium (Ba), Thallium (Tl), and Lead (Pb). The observed VIFs ranged from 1.257 to 2.965, all of which were substantially below the conventional cutoffs of 5 and 10.

Table S6. Quartile-based associations between blood element concentrations and depressive symptoms in multivariable logistic regression models.

elements	Q1	Q2		Q3		Q4		Continuous	
	reference	OR (95% CI)	*P* value	OR (95% CI)	*P* value	OR (95% CI)	*P* value	OR (95% CI)	*P* value
Mg	reference	0.535 (0.338-0.837)	0.007	0.466 (0.286-0.749)	0.002	0.489 (0.294-0.801)	0.005	0.148 (0.050-0.434)	0.001
Al	reference	0.820 (0.472-1.417)	0.478	1.444 (0.856-2.459)	0.171	1.940 (1.151-3.319)	0.014	1.560 (1.126-2.174)	0.008
V	reference	0.728 (0.442-1.194)	0.210	1.324 (0.831-2.120)	0.239	1.246 (0.768-2.030)	0.373	1.101 (0.950-1.294)	0.222
Cr	reference	1.427 (0.863-2.378)	0.168	1.568 (0.958-2.595)	0.076	1.258 (0.749-2.133)	0.389	1.044 (0.952-1.153)	0.371
Mn	reference	0.655 (0.393-1.080)	0.100	0.832 (0.510-1.352)	0.458	1.213 (0.752-1.967)	0.430	1.174 (0.814-1.735)	0.404
Fe	reference	0.838 (0.528-1.324)	0.450	0.672 (0.408-1.094)	0.113	1.104 (0.687-1.773)	0.682	0.810 (0.271-2.430)	0.706
Co	reference	0.946 (0.578-1.548)	0.825	1.005 (0.618-1.637)	0.984	1.214 (0.764-1.940)	0.414	1.015 (0.942-1.100)	0.707
Ni	reference	0.572 (0.344-0.940)	0.029	0.694 (0.432-1.110)	0.128	0.758 (0.470-1.223)	0.256	0.869 (0.741-1.022)	0.086
Cu	reference	1.350 (0.827-2.223)	0.232	1.061 (0.635-1.780)	0.820	1.445 (0.884-2.387)	0.145	2.937 (0.828-10.549)	0.097
Zn	reference	0.948 (0.602-1.487)	0.815	0.942 (0.590-1.498)	0.802	0.882 (0.540-1.432)	0.612	0.974 (0.507-1.881)	0.937
As	reference	0.658 (0.401-1.072)	0.094	0.815 (0.508-1.306)	0.396	1.060 (0.668-1.683)	0.805	1.068 (0.867-1.344)	0.787
Se	reference	1.390 (0.892-2.179)	0.147	0.958 (0.593-1.543)	0.859	0.686 (0.416-1.122)	0.135	0.841 (0.570-1.246)	0.379
Sr	reference	0.965 (0.572-1.626)	0.892	1.156 (0.702-1.917)	0.570	1.787 (1.101-2.938)	0.020	1.890 (1.278-2.791)	0.001
Mo	reference	1.395 (0.821-2.402)	0.222	1.449 (0.853-2.500)	0.175	1.491 (0.873-2.592)	0.149	1.108 (0.816-1.551)	0.533
Cd	reference	0.848 (0.523-1.371)	0.502	0.975 (0.611-1.559)	0.916	0.743 (0.433-1.266)	0.277	0.887 (0.727-1.093)	0.248
Ba	reference	1.267 (0.751-2.154)	0.377	1.322 (0.788-2.239)	0.293	1.932 (1.172-3.240)	0.011	1.623 (1.081-2.427)	0.019
Tl	reference	0.658 (0.404-1.060)	0.088	0.781 (0.489-1.241)	0.298	0.821 (0.522-1.289)	0.392	0.940 (0.871-1.017)	0.116
Pb	reference	0.737 (0.447-1.211)	0.228	1.268 (0.795-2.035)	0.320	0.868 (0.519-1.448)	0.589	1.002 (0.918-1.099)	0.967

Notes: OR, odds ratio; CI, confidence interval; Q, quartile. Q1 was used as the reference category. Models were adjusted for age, sex, educational level, income, BMI, marital status, living status, drinking, smoking, hypertension, diabetes, and coronary heart disease.

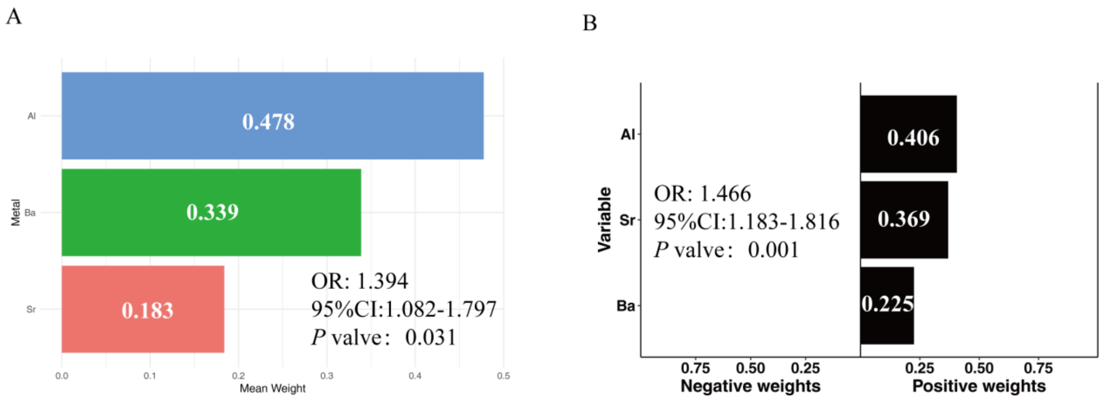

Figure S3. Weights estimated from the weighted quantile sum (WQS) and quantile g-computation (qgcomp) models for depressive symptoms. Al, Sr, and Ba were included in the mixture models.
